# Ivermectin Synergizes with Modulated Electro-hyperthermia and Improves Its Anticancer Effects in a Triple-Negative Breast Cancer Mouse Model

**DOI:** 10.1021/acsptsci.4c00314

**Published:** 2024-07-17

**Authors:** Kenan Aloss, Pedro Henrique Leroy Viana, Syeda Mahak Zahra Bokhari, Nino Giunashvili, Csaba András Schvarcz, Dániel Bócsi, Zoltán Koós, Zoltán Benyó, Péter Hamar

**Affiliations:** † Institute of Translational Medicine, Semmelweis University, Üllői út 26., Budapest 1085, Hungary; ‡ Department of Pharmacology and Pharmacotherapy, Semmelweis University, Budapest 1089, Hungary; § HUN-REN-SU Cerebrovascular and Neurocognitive Diseases Research Group, Tűzoltó utca 37-47., Budapest 1094, Hungary

**Keywords:** triple-negative breast cancer, modulated electro-hyperthermia, ivermectin, heat shock protein beta 1, apoptosis

## Abstract

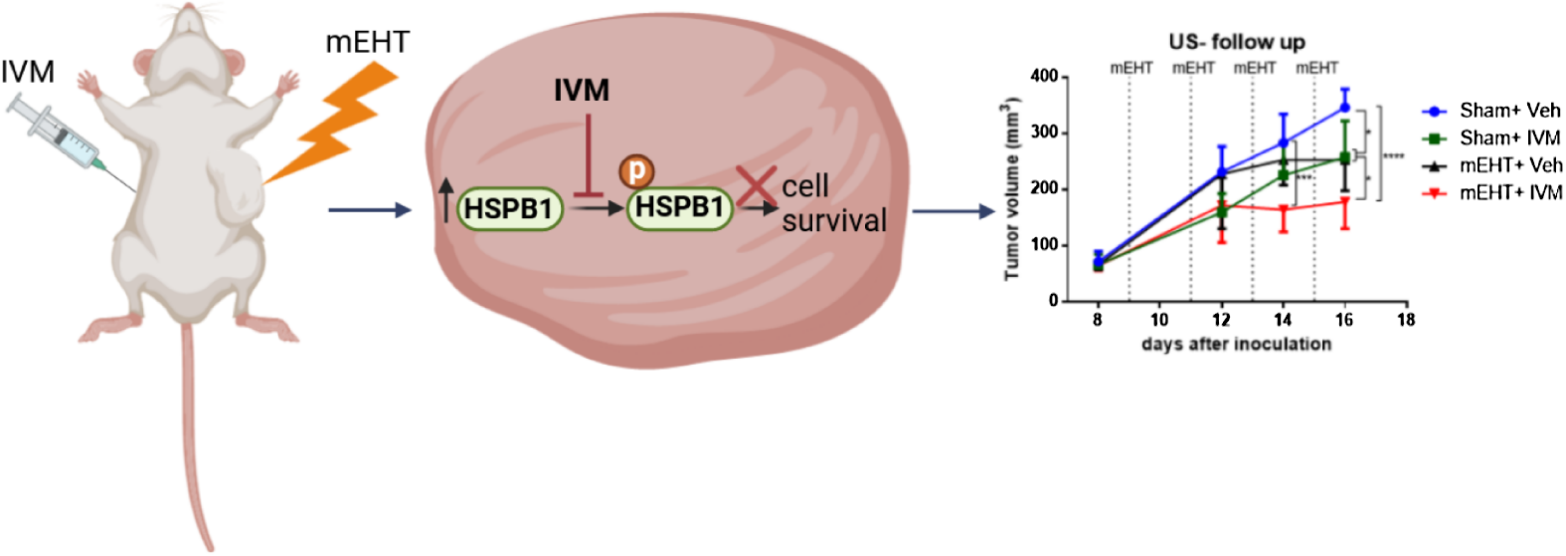

Triple-negative breast cancer (TNBC) is the most aggressive breast cancer subtype, with limited treatment options. Modulated electro-hyperthermia (mEHT) is a novel adjuvant cancer therapy that induces selective cancer damage. However, mEHT upregulates heat shock protein beta 1 (HSPB1), a cancer-promoting stress chaperone molecule. Thus, we investigated whether ivermectin (IVM), an anthelmintic drug, may synergize with mEHT and enhance its anticancer effects by inhibiting HSPB1 phosphorylation. Isogenic 4T1 TNBC cells were inoculated into BALB/c mice and treated with mEHT, IVM, or a combination of both. IVM synergistically improved the tumor growth inhibition achieved by mEHT. Moreover, IVM downregulated mEHT-induced HSPB1 phosphorylation. Thus, the strongest cancer tissue damage was observed in the mEHT + IVM-treated tumors, coupled with the strongest apoptosis induction and proliferation inhibition. In addition, there was no significant body weight loss in mice treated with mEHT and IVM, indicating that this combination was well-tolerated. In conclusion, mEHT combined with IVM is a new, effective, and safe option for the treatment of TNBC.

## 1 Introduction

Triple-negative breast cancer (TNBC) is a highly aggressive breast cancer type with a poor prognosis and accounts for approximately 15% of total breast cancer cases.^1^ TNBC cells lack the expression of the estrogen receptor (ER), progesterone receptor (PR), and human epidermal growth factor receptor-2 (HER2). Thus, presently available targeted therapies are not effective, which necessitates the need for alternative treatments.^2^

Heat shock protein beta 1 (HSPB1), also known as HSP27 in humans and HSP25 in rodents (HSP20 family), is a small molecular chaperone that prevents the denaturation of cellular proteins in response to different environmental stressors including elevated temperatures.^3^ The function of HSPB1 is regulated by post-translational phosphorylation, which increases its affinity for destabilized proteins.^4,5^ HSPB1 is phosphorylated at serine (Ser) residues Ser-15, Ser-78, and Ser-82 in humans and Ser-15 and Ser-86 in rodents.^6^ Upon heat stress, HSPB1 is phosphorylated and shuffles from the cytoplasm to the nucleus to protect the nuclear structure and functions as a molecular chaperone that promotes the correct folding of other proteins.^7−10^ The chaperone activity of phosphorylated HSPB1 includes the improvement of the stability and recovery of actin filaments, which are important for cell survival during stress.^8,11^ Expression of HSPB1 is correlated with a poor clinical outcome in multiple human cancers as HSPB1 promotes cancer cell proliferation and protects cancer cells from apoptosis.^12^ Thus, the inhibition of HSPB1 phosphorylation might inhibit survival pathways and improve cancer cell death during heating.

Ivermectin (IVM) is an FDA-approved antiparasitic drug that exerts its effects by activating invertebrates-specific glutamate-gated chloride channels.^13^ IVM can also interact with mammalian neuronal receptors, such as GABA receptors; however, its poor permeability across the blood–brain barrier contributes to its safety profile in healthy humans at clinical doses (200 μg/kg).^14,15^ However, IVM overdosing can cause several side effects such as neurotoxicity, hypotension, and respiratory failure.^16,17^ Several studies have reported promising anticancer effects for IVM in different cancer models.^18−20^ IVM suppresses cancer cell proliferation through the induction of cell cycle arrest.^21,22^ In addition, IVM induces cancer cell death mainly via the mitochondrial pathway of apoptosis, resulting in the activation of caspase 3, a key mediator of apoptosis.^21,23^ Recently, IVM has been demonstrated to inhibit the activity of HSPB1 by binding its phosphorylation pocket, preventing its phosphorylation and interaction with misfolded proteins.^24^ However, the possibility of using IVM to inhibit hyperthermia (HT)-induced HSPB1 and thus improve HT efficacy has not been investigated in vivo.

Modulated electro-hyperthermia (mEHT) is a noninvasive adjuvant cancer treatment approved for clinical use in different cancer types.^25−28^ mEHT uses an amplitude-modulated, 13.56 MHz radiofrequency electromagnetic field by capacitive coupling between two electrodes surrounding the cancer.^29,30^ Due to the difference in bioelectrical properties between cancerous and healthy tissues,^31,32^ the electromagnetic field energy is absorbed mainly by cancer tissues, resulting in cancer-specific tissue damage.^33,34^ At the cellular level, mEHT generates heat, leading to cellular stress and upregulation of HSPs.^35^ We previously demonstrated enhanced anticancer effects of mEHT by inhibiting HSP70 in vitro^36^ and heat shock factor 1 (HSF1) in vivo,^37^ utilizing the small molecule inhibitors of the heat shock response central regulator (i.e., HSF1): quercetin and KRIBB11, and by knocking down HSF1 production of the tumor cells with a CRISPR/Cas9 construct.^36^ However, quercetin and its derivatives enhance HSPB1 phosphorylation that supports cancer cell survival.^38^ Moreover, KRIBB11 has not yet reached the clinical trial phase. Thus, the inhibition of mEHT-induced HSPs by FDA-approved drugs is justified to increase the clinical relevance of this combinational therapy.

Previously, our group revealed that mEHT upregulated HSPB1 24 h after the treatment.^39^ Therefore, in the present study, we investigated whether IVM might enhance the anticancer effects of mEHT by reducing the phosphorylation of HSPB1 in 4T1-bearing mice. The current study demonstrated that IVM augmented mEHT-induced cancer cell death. Furthermore, IVM downregulated the phosphorylated form of HSPB1. Consequently, a combination of mEHT and IVM enhanced apoptosis and attenuated proliferation of TNBC compared to monotherapy with IVM or mEHT. In addition, the applied IVM dose was equivalent to the clinically used IVM doses, indicating the clinical relevance of this combinational therapy.

## 2 Experimental Section

### 2.1 Cell Culture

4T1 (RRID:CVCL_0125) triple-negative breast cancer cells were obtained from the Lieberman Laboratory (Harvard University, Boston, MA, USA). The status of ER, PR, and HER-2 was confirmed in 4T1 cells and tumors by PCR (Figure S1). 4T1 cells were grown in Dulbecco’s modified essential medium (DMEM 4.5 g/L glucose, without l-glutamine, Capricorn Scientific, Ebsdorfergrund, Germany) supplemented with l-glutamine 200 mM (Capricorn Scientific, Ebsdorfergrund, Germany), 10% fetal bovine serum (FBS- EuroClone S.p.A., Pero, Italy, Cat. No. ECS0180L), and 10% penicillin-streptomycin mixture (Capricorn Scientific, Ebsdorfergrund, Germany). All cell lines were subjected to routine screening for the mycoplasma. In addition, all experiments were performed using cells confirmed to be mycoplasma-free. Within the last three years, the authenticity of the cell lines was confirmed through repeated evaluations, which involved comparing newly obtained data with established databases and reference panels. This process ensures the continuous validation of the identities of the cell lines.

### 2.2 In Vivo Model

**Figure 1:**
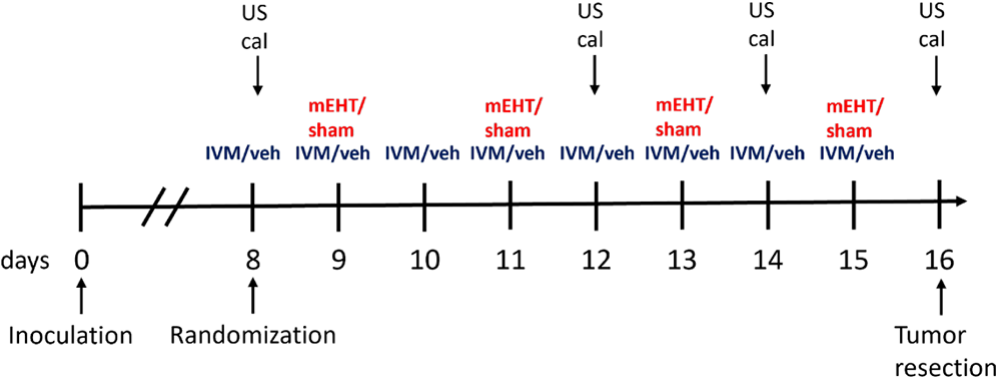
Schedule of the tumor growth inhibition study. Mice were injected daily with 7.5 mg/kg IVM in mEHT + IVM and sham + IVM groups or the equivalent amount of veh in mEHT + veh and sham + veh (control) groups. All mice were treated either with mEHT or sham on days 9, 11, 13, and 15. Tumor volume was measured using US and cal on days 8, 12, 14, and 16. mEHT: modulated electro-hyperthermia, IVM: ivermectin, veh: vehicle, US: ultrasound, cal: caliper.

Female BALB/c mice, aged between 6 and 8 weeks, were bred in the specific pathogen-free (SPF) animal facility at the Department of Oncology, Semmelweis University. Mice were provided with unrestricted access to standard rodent chow and water and subjected to a 12 h dark/12 h light cycles. Prior to cancer cell inoculation, mice were anesthetized using 5% concentration of isoflurane (Baxter International Inc., Deerfield, IL, USA) for induction, followed by maintenance anesthesia at 1.5–2% with compressed airflow set at 0.4–0.6 L/min. 1 × 10^6^ 4T1 cells in 50 μL phosphate buffered saline (PBS) were orthotopically inoculated into the fat pad of the fourth mammary gland of each mouse using a Hamilton syringe. Eight days after inoculation, tumor volume was measured by ultrasound (US) and digital caliper as previously described.^36^ Mice were randomized according to tumor volume into four groups: sham + vehicle, sham + IVM, mEHT + veh, and mEHT + IVM (*n* = 5–7). The number of mice per group was based on our experience from previous studies with mEHT. Their tumor volume and body weight were monitored until study termination. Mice were euthanized by cervical dislocation at 24 h after the last treatment, and tumors were resected and weighted (Figure 1). Half of the tumor was immersed in a 4% formaldehyde solution (Molar Chemicals Ltd., Halásztelek, Hungary) and later subjected to histological processing. The remaining tumor tissue was preserved in liquid nitrogen at −80 °C for subsequent molecular analysis.

### 2.3 mEHT and IVM In Vivo Treatment

Mice underwent four mEHT treatments, with 48 h intervals between the treatments (Figure 1). mEHT treatment was done using a LabEHY 200 device (Oncotherm kft., Budaörs, Hungary) as described previously.^36,40^ Briefly, mice under 5% isoflurane anesthesia were placed on a heating pad (i.e., the lower electrode), and the upper electrode was positioned on the tumor. mEHT was conducted at 0.7 ± 0.3 W for 30 min in a temperature-controlled manner, following a 3 min warm-up period. The temperature of the tumor, rectum, heating pad, and room was monitored by using optical temperature sensors (Oncotherm Ltd., Budaörs, Hungary). Tumor temperature was maintained at 42 °C during the 30 min of treatment. In the sham group, mice were subjected to the same conditions as the mEHT-treated mice (i.e., heating pad temperature and the upper electrode position); however, the electromagnetic field was not turned on (no mEHT).

IVM-treated mice received 7.5 mg/kg IVM (#HY-15310, MedChemExpress, NJ, USA), administered daily via an intraperitoneal injection (Figure 1). IVM was dissolved in a vehicle (veh) composed of 5% dimethyl sulfoxide (DMSO), 40% polyethylene glycol (PEG) 300, 5% Tween-80, and 50% saline. In the sham and mEHT groups, mice received equivalent amounts of the vehicle.

The Bliss independence principle was used to determine whether the interaction between mEHT and IVM was synergistic or additive. The Bliss-predicted inhibition rate was calculated based on the individual effects of mEHT and IVM on tumor weight compared to sham + veh. The Bliss-predicted inhibition rate was compared to the observed inhibitory effect of mEHT + IVM. The interaction was considered synergistic when the observed effect exceeded the predicted (additive) effect.^41^

### 2.4 qPCR

Tumor tissues were mixed with 1 mL of TRIzol reagent (Molecular Research Center LLC, Cincinnati, Oh, USA) and disrupted mechanically using TissueRuptor (Qiagen, Hilden, Germany). RNA was extracted from the tissue homogenate and purified using the TRIzol method as described previously.^42^ A High-Capacity cDNA Reverse Transcription kit (Applied Biosystems, Carlsbad, CA, USA) was used to reverse-transcribe the extracted RNA. The resulting cDNA was used as a template for RT-PCR. Messenger RNA was detected in the samples using the SsoAdvanced Universal SYBR Green supermix and the CFX96 Touch Real-Time PCR Detection system (Bio Rad, Hercules, CA, USA). HSPB1 expression was normalized to GAPDH. Table 1 summarizes the primers used in this study.

**Table 1:** Primers Used for RT-PCR

gene symbol	gene name	primer pairs
*GAPDH*	glyceraldehyde-3-phosphate dehydrogenase (*Mus musculus*)	fwd: CTCCCACTCTTCCACCTTCG
		rev: GCCTCTCTTGCTCAGTGTCC
*HSPB1*	heat shock protein beta 1 (*Mus musculus*)	fwd: AAG GAA GGC GTG GTG GAG AT
		rev: TTC GTC CTG CCT TTC TTC GT

### 2.5 Western Blot

Total protein was isolated from tumor samples using TRIzol reagent (Molecular Research Center lnc., Ohio, USA) according to the manufacturer’s instructions. Twenty micrograms of the isolated protein were loaded per well and separated on 12% SDS-PAGE. The separated proteins were transferred onto a polyvinylidene difluoride (PVDF) membrane (#1704156, Bio-Rad, Hercules, CA, USA). The membrane was cut into two to probe the same membrane for two proteins simultaneously. Blocking and antibody dilutions were performed in Tris-buffered saline, supplemented with 5% skim milk and 0.05% Tween 20. The membrane was incubated with a primary antibody specific for HSPB1, phosphorylated-HSPB1 (p-HSPB1), cleaved caspase 3 (cC3), or β-actin overnight at 4 °C (Table 2). After being washed, the membrane was incubated with a horseradish peroxidase (HRP)-conjugated secondary antibody for an hour. Detection and visualization of the chemiluminescent signal were conducted by an ECL Prime Western Blotting Detection reagent (#RPN2232, Cytiva, MA, USA) and an Imager CHEMI premium (VWR, Radnor, PA, USA). The expression of proteins of interest was analyzed by ImageJ software and normalized to β-actin.

**Table 2:** Antibodies Used for Western Blot (WB) and Immunohistochemistry (IHC)^a^

antibody	type	reference no.	method	dilution	vendor
β-actin	mouse, mAb	#ab6276	WB	1:5000	Abcam
HSPB1	mouse, mAb	# sc-13132	WB	1:1000	Santa Cruz
p-HSPB1 (Ser86)	rabbit, pAb	#44-536G	WB	1:1000	Invitrogen
	rabbit, pAb	#2401	IHC	1:50	Cell Signaling
cC3	rabbit, mAb	##9664	WB	1:1000	Cell Signaling
			IHC	1:1600	
Ki67	rabbit, mAb	#MA5-14520	IHC	1:50	Invitrogen
antirabbit (secondary)	goat, pAb	#A27036	WB	1:5000	Invitrogen
antimouse (secondary)	goat, pAb	#A28177	WB	1:5000	Invitrogen

a HSPB1: heat shock protein beta 1, p-HSPB1: phosphorylated-HSPB1, Ser86: serine-86 residue: the site of the phosphorylation, cC3: cleaved caspase 3, Ki67: Kiel-67 proliferation marker, mAb: monoclonal antibody, and pAb: polyclonal antibody.

### 2.6 Histopathology and Immunohistochemistry

Tumor tissue samples were fixed in 10% neutral-buffered formalin before being embedded in paraffin (FFPE). FFPE tumors were cut into 2.5 μm sections, mounted on glass slides, and kept in a thermostat set to 65 °C for 1 h. Tumor sections were deparaffinized and rehydrated for hematoxylin-eosin (H&E) staining and immunohistochemistry (IHC). H&E staining was performed as described in detail earlier.^40^ H&E-stained tumor sections were scanned and evaluated digitally using the Slide Viewer software (v.2.6, 3DHISTECH, Budapest, Hungary). The damaged tumor area was annotated from the viable tumor area under high magnification. Tumor destruction ratio (TDR) % was obtained by dividing the damaged area by the whole tumor area as described previously.^43^

IHC for p-HSPB1, cC3, and Kiel-67 (Ki67) was conducted with a Polymer-Peroxidase system (Histols, Histopathology Ltd., Pécs, Hungary) as described in detail earlier.^40^ The stained slides were scanned, and the positively stained area was analyzed using the QuantCenter module of Slide Viewer software. The expression of p-HSPB1 and cC3 staining was estimated in the whole tumor area by masking the specific dark brown staining with green and indicated as the relative mask area. Ki67-positive nuclei were counted digitally in the annotated viable area.

### 2.7 Statistical Analysis

Data are presented as the mean ± SEM. The statistical analysis was performed with GraphPad Prism software (v.6.01; GraphPad Software, Inc., La Jolla, CA, USA). Changes in tumor volume and body weight were compared between groups by two-way ANOVA and Tukey’s post hoc test. Changes in tumor weight and protein expression were analyzed by one-way ANOVA and Tukey’s post hoc. The null hypothesis was rejected if *p* < 0.05.

## 3 Results

### 3.1 IVM Synergistically Enhanced Tumor Growth Inhibition Achieved by mEHT

**Figure 2:**
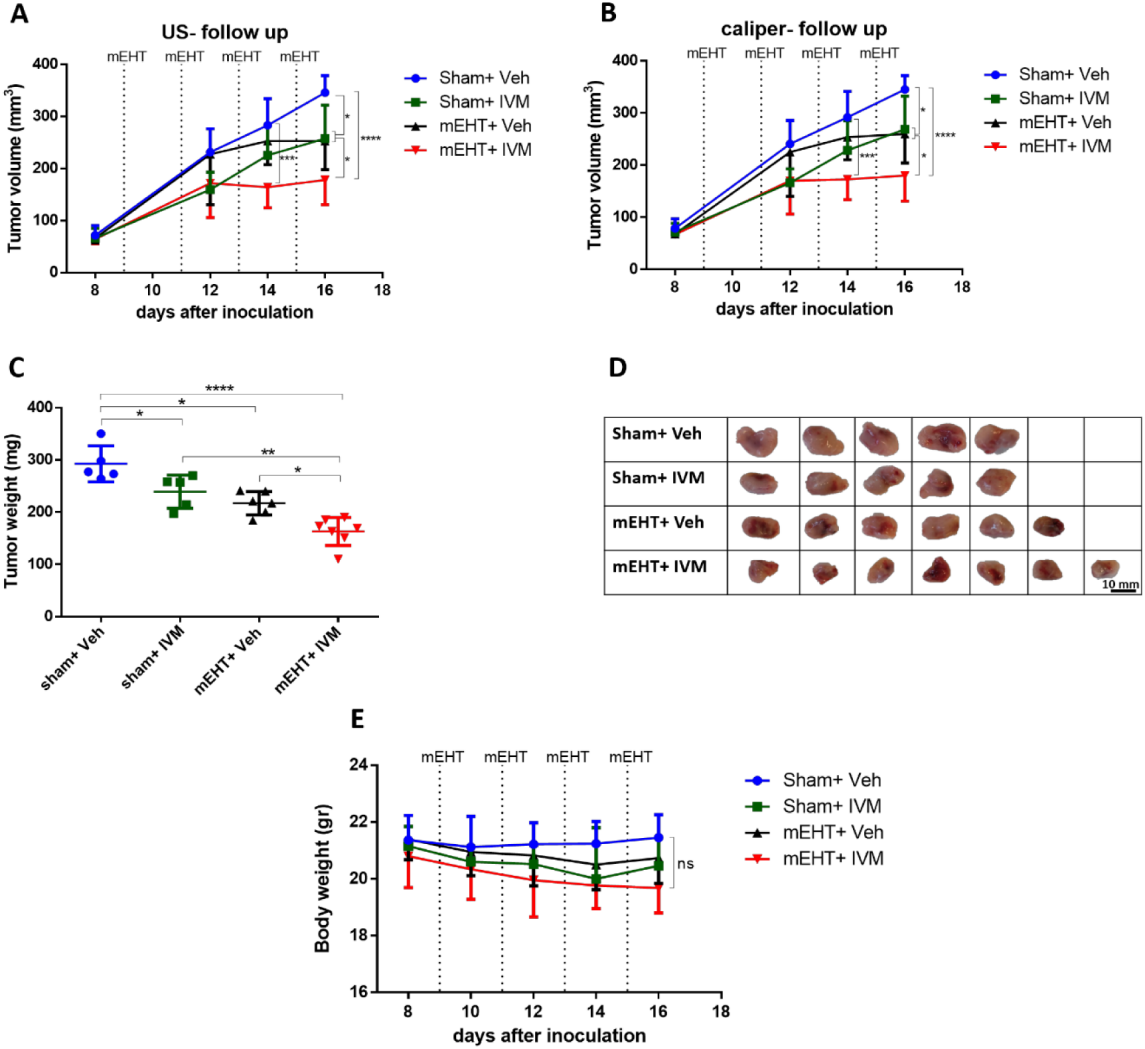
Effect of different treatments on tumor growth and body weight in 4T1-bearing mice. Veh: vehicle, mEHT: modulated electro-hyperthermia, and IVM: ivermectin. (A) Ultrasound (US) and (B) digital caliper data after four mEHT treatments (dotted lines). (C) Tumor weight. (D) Scale images of the excised tumors. Each row shows all tumors in that group. (E) Body weight. Mean ± SEM, (A, B, E) two-way ANOVA and Tukey’s post hoc test, and (C) one-way ANOVA and Tukey’s post hoc test. *n* = 5–7/group, ns: not significant, *p < 0.05, **p < 0.01, and ****p < 0.0001.

Sham + veh tumors grew constantly during the study. The growth of mEHT + IVM-treated tumors decelerated after the third treatment. Consequently, the size of (mEHT + IVM)-treated tumors was significantly smaller than the size of sham + veh tumors after the third treatment (Figure 2 A,B). Upon study termination, sham + veh tumors were the biggest. Both IVM- and mEHT monotherapy-treated tumors were significantly smaller than sham + veh tumors. However, the tumors receiving (mEHT + IVM) combination treatment were the smallest (Figure 2 C,D).

Based on tumor weight means (Figure 2C), the tumor inhibition rates of IVM and mEHT monotherapies compared to the sham + veh group were 18.2% and 24.3%, respectively. Thus, the Bliss-predicted inhibition rate was 42.5%. The mEHT + IVM treatment demonstrated a tumor inhibition rate of 44.3% compared to sham + veh, indicating synergism between mEHT and IVM.

Body weight was not reduced significantly in any of the study groups, suggesting a lack of severe toxicity. Sham + veh-treated mice gained weight slightly during the study. Mice treated with IVM, mEHT, or mEHT + IVM experienced a slight body weight loss after the third treatment. The body weight loss did not exceed 6% in any of these groups. By the end of the study, there was no significant difference in body weight loss between the groups (Figure 2E).

### 3.2 mEHT Upregulated HSPB1 at 24 h after the Treatment

3.2

**Figure 3:**
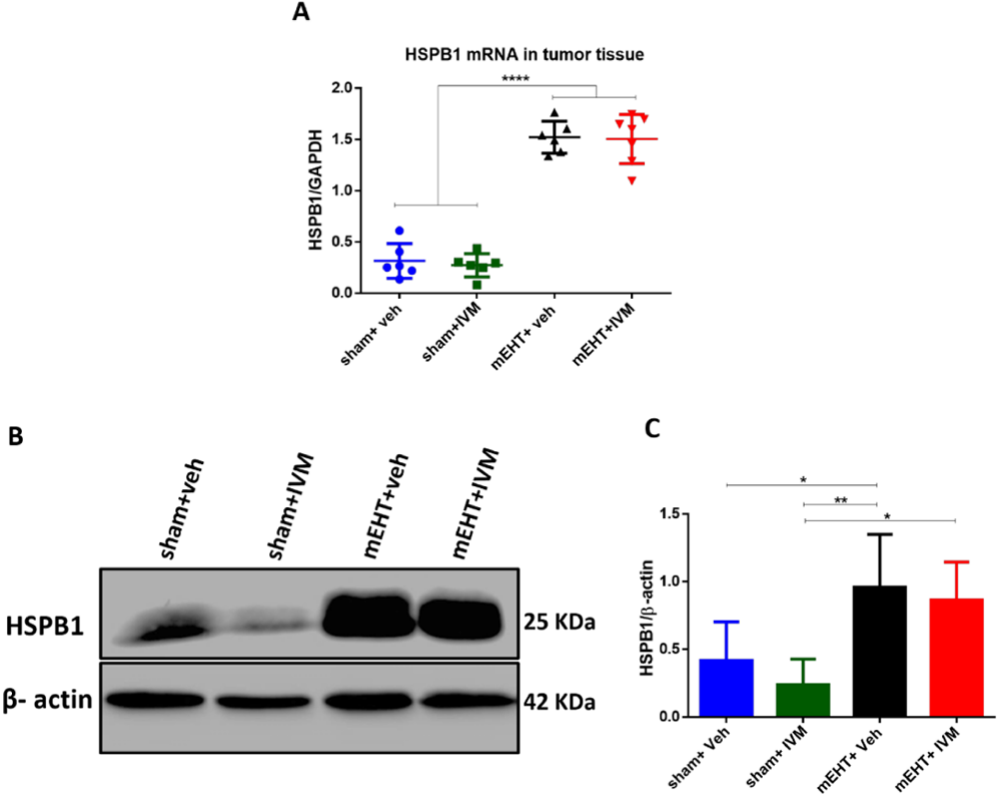
HSPB1 mRNA and protein expression 24 h after the last treatment. (A) mRNA expression of HSPB1 in the tumor. (B) Representative images of Western blot. (C) Quantification of HSPB1 protein expression relative to β-actin. Mean ± SEM and (A, C) one-way ANOVA and Tukey’s post hoc test. *n* = 5–7/group, *p < 0.05, **p < 0.01, ***p < 0.001, and ****p < 0.0001.

We observed low gene and protein expression of HSPB1 in tumors treated with sham + veh or sham + IVM 24 h after the last treatment. However, mEHT significantly upregulated HSPB1 gene and protein expression as compared to sham groups 24 h after the last treatment. Combining IVM with mEHT did not affect the gene or protein expression of HSPB1 (Figure 3A–C).

### 3.3 IVM Attenuated the Phosphorylation of HSPB1

**Figure 4:**
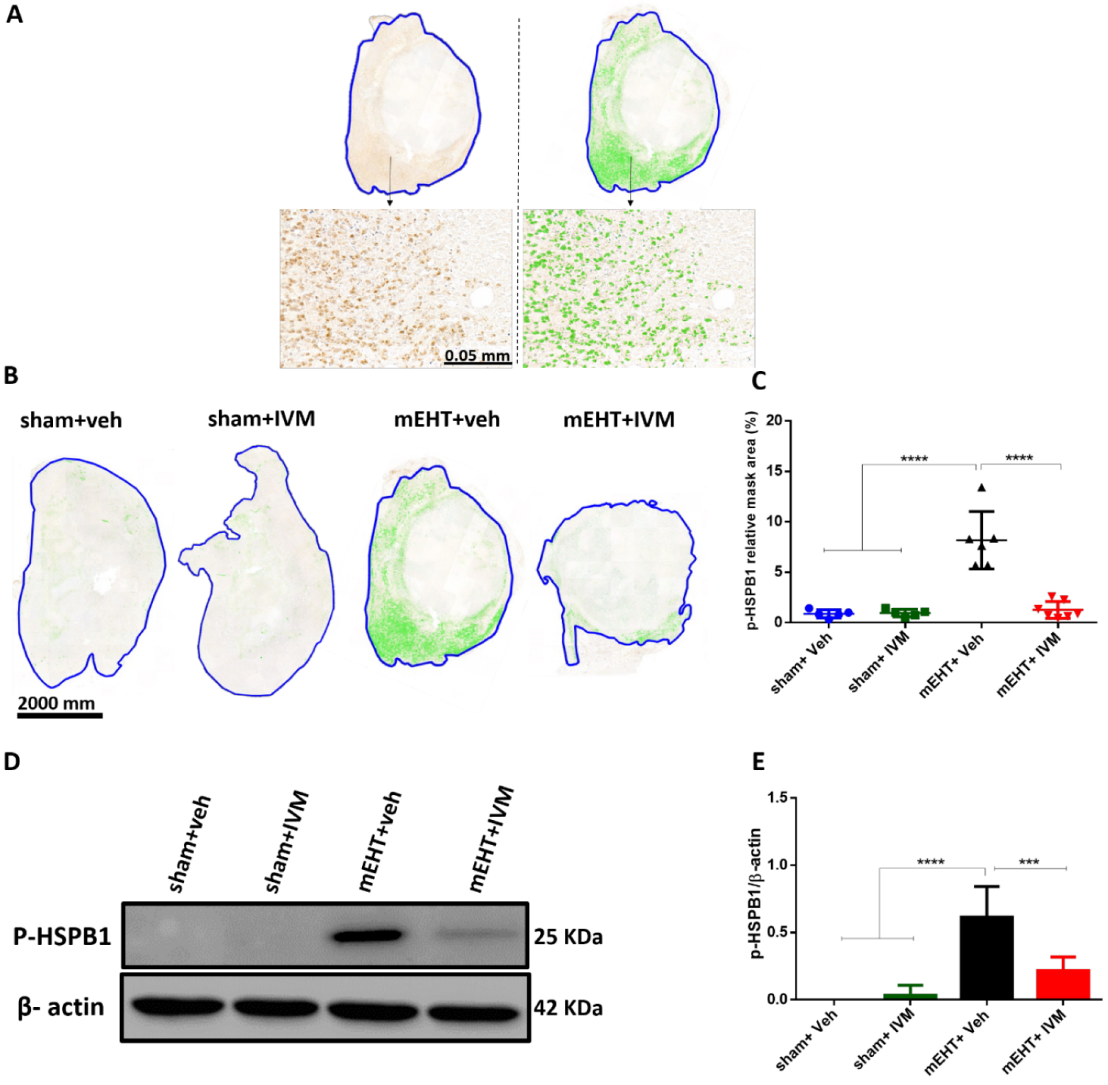
Expression of phosphorylated HSPB1 (p-HSPB1) 24 h after the last treatment. (A) Representative image depicting the digital evaluation of p-HSPB1 expression on an immunohistochemistry (IHC)-stained tumor section. p-HSPB1-specific staining (dark brown) was masked with green for quantification. (B) Representative images of p-HSPB1-immunostained tumor sections at 0.9 × magnification. (C) Quantification of relative p-HSPB1 staining in the tumor expressed as a relative mask area. (D) Representative images of Western blot. (E) Quantification of p-HSPB1 protein expression relative to β-actin. Mean ± SEM and (C, E) one-way ANOVA and Tukey’s post hoc test. *n* = 5–7/group, ***p < 0.001, and ****p < 0.0001.

The effect of IVM on HSPB1 phosphorylation was tested by studying the expression of p-HSPB1 in the tumor tissue 24 h after the last treatment. On IHC-stained tumor sections, p-HSPB1-positive areas were stained dark brown, which were masked with green for quantification (Figure 4A). IHC demonstrated weak p-HSPB1 staining in tumors treated with sham + veh or sham + IVM. p-HSPB1 staining was stronger in the mEHT group compared to that in sham + veh and sham + IVM. IVM could significantly reduce the mEHT-induced p-HSPB1 staining compared to mEHT alone (Figure 4B,C). Consistent with the IHC results, Western blot analysis demonstrated that mEHT + IVM- tumors had a significantly lower p-HSPB1 expression than tumors treated with mEHT monotherapy (Figure 4 D,E).

### 3.4 IVM Augmented mEHT-Induced Tumor Tissue Destruction

**Figure 5:**
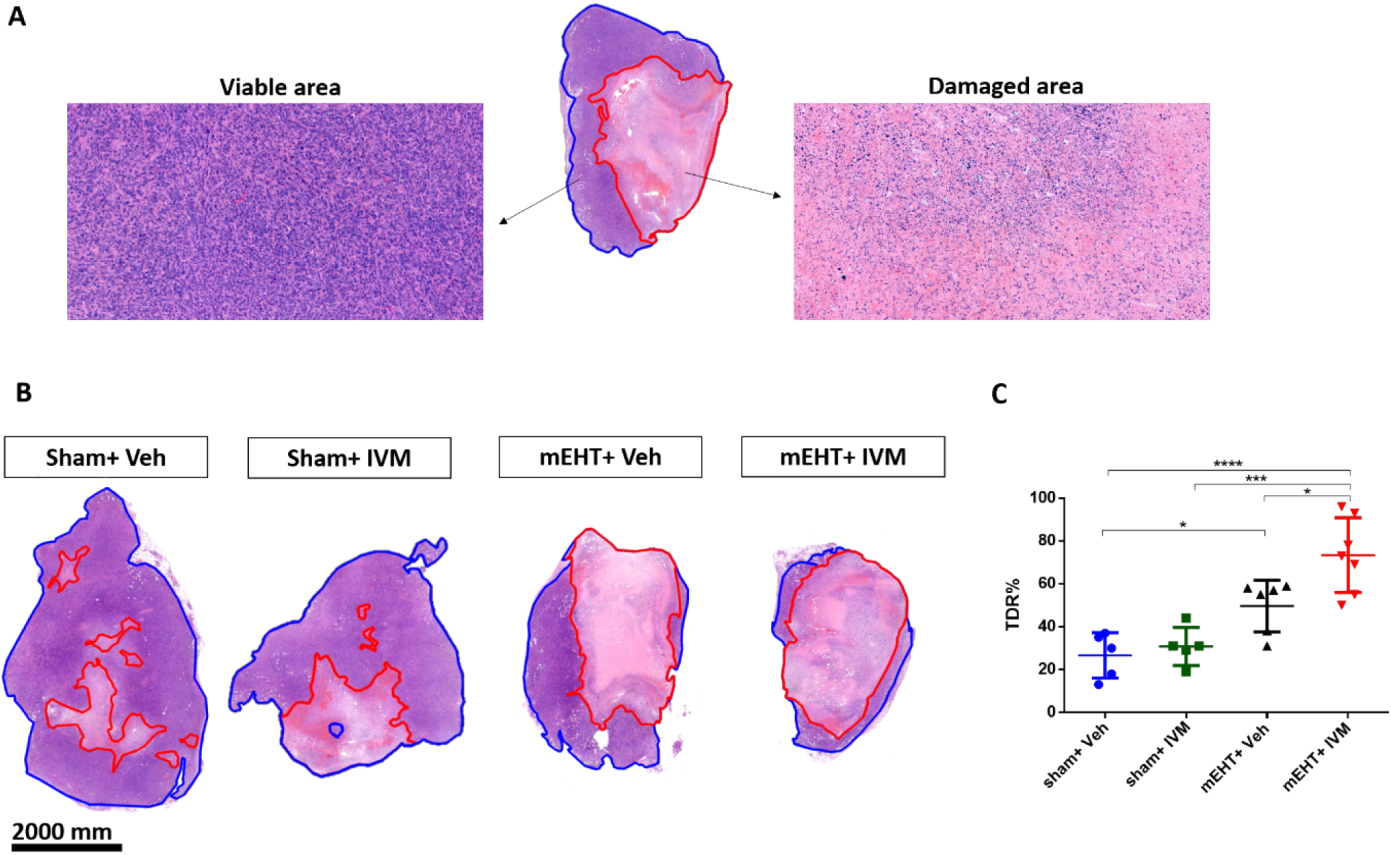
Tumor destruction ratio (TDR) 24 h after the last treatment. (A) The difference between damaged and viable tumor areas on hematoxylin-eosin (H&E)-stained tumor sections. Damaged (red) and whole tumor (blue) areas were annotated. (B) Representative images of (H&E)-stained tumor sections at 0.9 × magnification. (C) TDR (%). Data are mean ± SEM and (C) one-way ANOVA and Tukey’s post hoc test. *n* = 5–7/group, *p < 0.05, ***p < 0.001, and ****p < 0.0001.

Cancer cell damage was estimated on H&E-stained tumor sections by calculating the TDR. The TDR was calculated by dividing the damaged area by the whole tumor area and expressed as %. The damaged tumor tissue was paler than the viable tissue on H&E-stained tumor sections (Figure 5A). Small, damaged areas were observed in sham + veh tumors. IVM alone could not induce TDR significantly compared to sham + veh. mEHT monotherapy induced stronger TDR as compared to sham + veh. However, mEHT + IVM induced the strongest TDR (Figure 5B,C).

### 3.5 IVM Improved Caspase-Dependent Apoptosis Induced by mEHT

**Figure 6:**
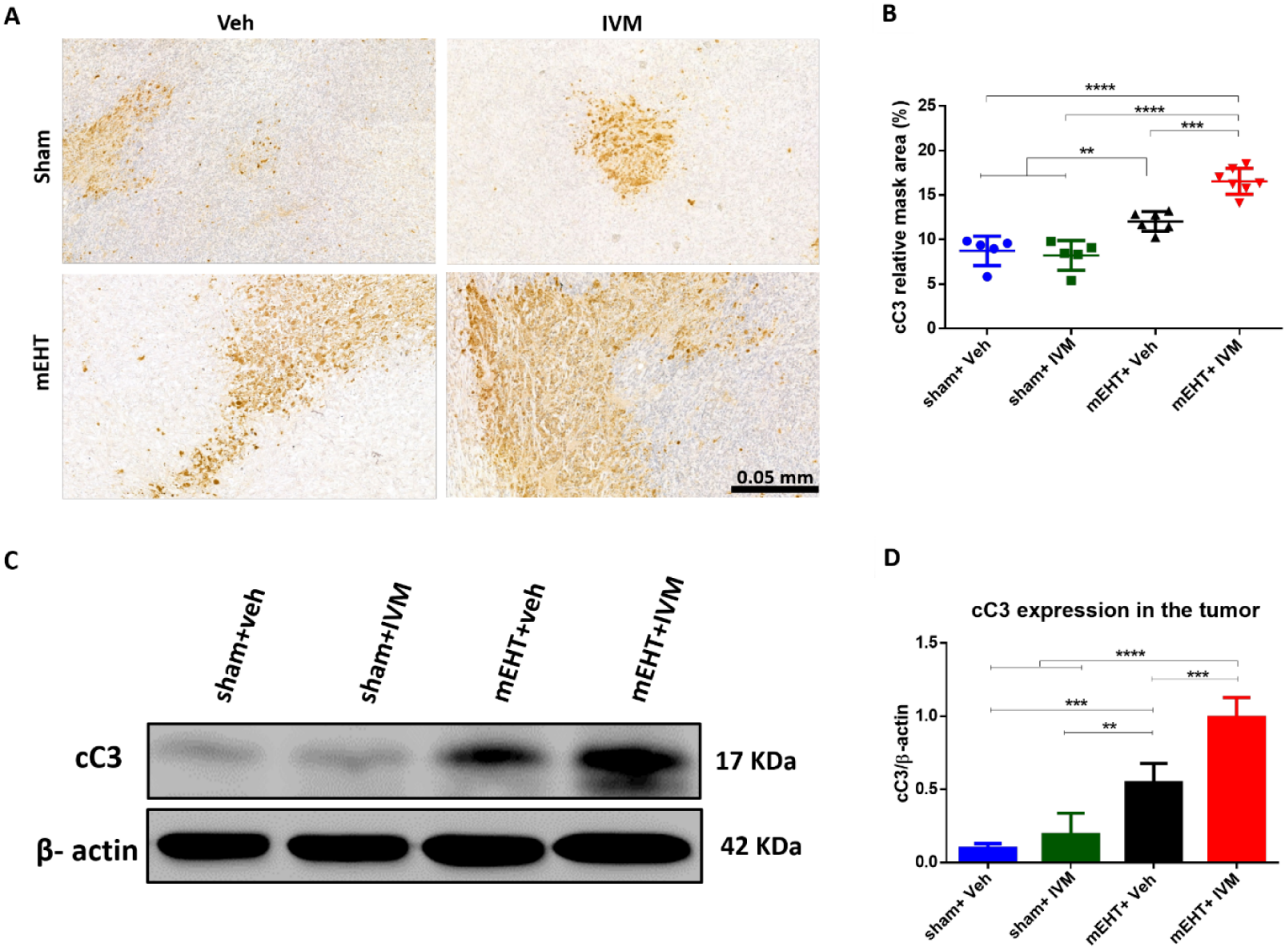
Expression and localization of cleaved caspase 3 (cC3) 24 h after the last treatment. (A) cC3-immunostained tumor sections at 40 × magnification. The cC3-positive area was stained with brown. (B) Quantification of relative cC3 staining in tumors indicated as a relative mask area. (C) Representative images of Western blot. (D) Quantification of cC3 protein expression relative to β-actin. Data are mean ± SEM and (B, D) one-way ANOVA and Tukey’s post hoc test. *n* = 5–7/group, **p < 0.01, ***p < 0.001, and ****p < 0.0001.

Apoptosis was evaluated 24 h after the last treatment by measuring cC3 expression in tumor tissues using IHC and Western blot. cC3 staining appeared dark brown on the IHC-stained tumors (Figure 6A). Sham + veh-treated tumors had small cC3-positive areas, demonstrating a lack of significant apoptosis. IVM alone could not induce cC3 significantly compared to sham + veh. mEHT-treated tumors had larger cC3-positive areas than sham + veh- or sham + IVM-treated tumors. However, cC3 staining was the most intensive in mEHT + IVM-treated tumors (Figure 6A,B). Similarly, Western blot demonstrated the strongest cC3 protein expression in mEHT + IVM-treated tumors (Figure 6C,D).

### 3.6 IVM Combined with mEHT Alleviated Tumor Cell Proliferation

**Figure 7:**
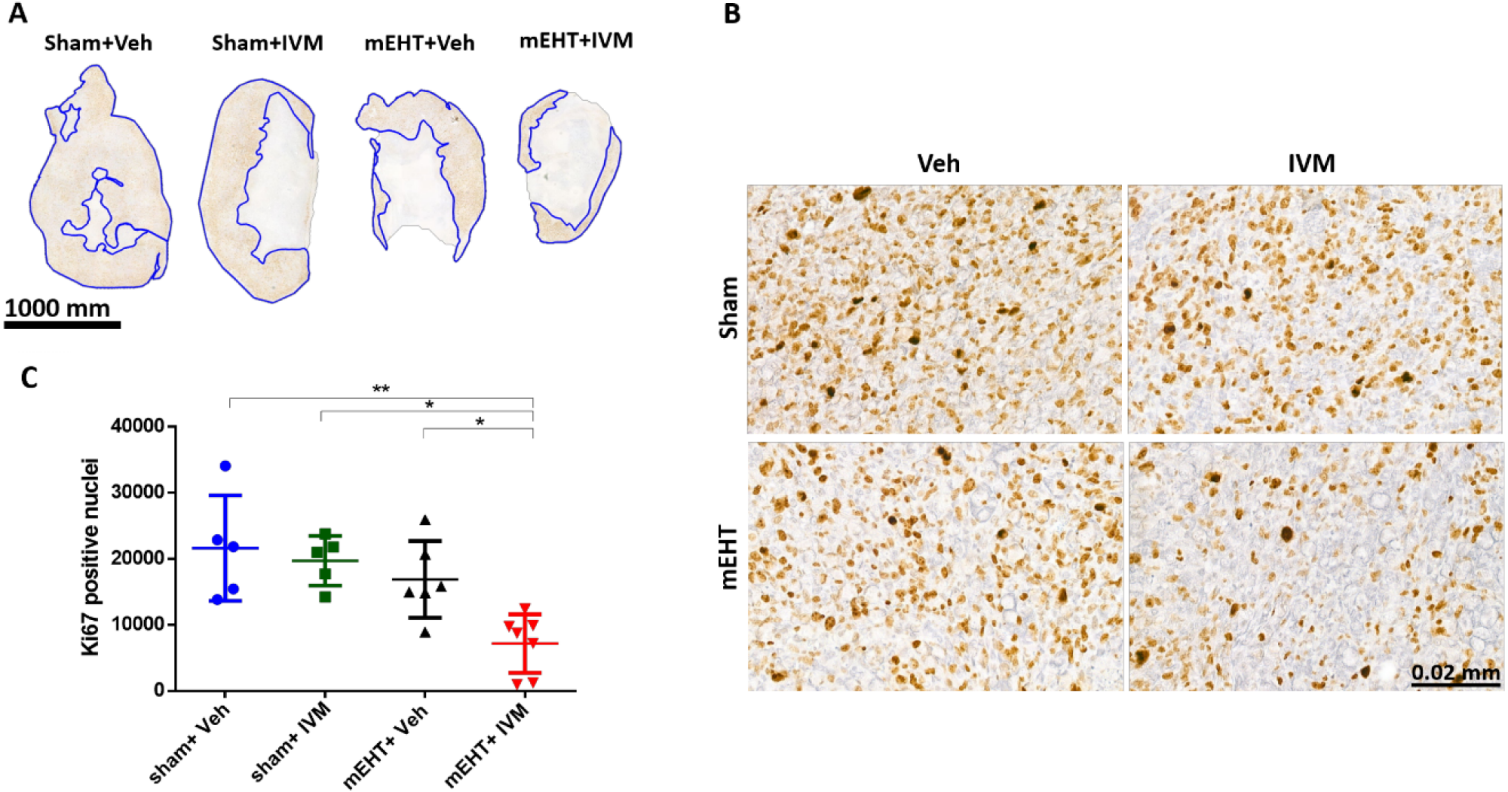
Effect of various treatments on tumor cell proliferation 24 h after the last treatment. Representative images of Ki67-stained tumor sections at 0.7 × magnification (A) and 40 × magnification (B). Ki67^+^ nuclei were stained with brown and counted in the viable tumor area (blue annotated). (C) Quantification of the Ki67^+^ nuclei in the tumor. Data are mean ± SEM and (C) one-way ANOVA and Tukey’s post hoc test. *n* = 5–7/group, *p < 0.05, and **p < 0.01.

The proliferation of cancer cells was studied by evaluating the expression of the Ki67 proliferation marker in the tumor at 24 h after the last treatment using IHC and Western blot. Ki67-positive (Ki67^+^) nuclei were stained brown in the annotated viable areas of the tumor (Figure 7A,B). The highest number of Ki67^+^ nuclei was in the (sham + veh)- and (sham + IVM)-treated tumors. mEHT could not reduce the number of Ki67^+^ nuclei as compared to sham + vehicle or sham + IVM. However, tumors in the (mEHT + IVM) group had significantly fewer Ki67^+^ nuclei than tumors in all other groups (Figure 7C).

## 4 Discussion

In our previous studies, we demonstrated that mEHT-induced a heat shock response (HSR) with the upregulation of HSF1 and HSP70. The concomitant inhibition of this HSR improved the anticancer effects of mEHT.^36,37^ However, the drugs applied in those studies have limitations, such as the enhancement of HSPB1 phosphorylation (e.g., quercetin)^38^ and the lack of clinical application (e.g., KRIBB11).^37^ Thus, to improve the clinical translational potency of this strategy, inhibition of mEHT-induced HSPs by FDA-approved drugs is needed. Thus, in this study, we investigated for the first time the possibility of enhancing the anticancer effects of modulated electro-hyperthermia (mEHT) using the FDA-approved anthelmintic drug, ivermectin (IVM), with demonstrated HSPB1 phosphorylation inhibiting effects. A triple-negative breast cancer (TNBC) mouse model was chosen to test the mEHT + IVM combination due to the limited treatment options in this cancer type.

The present study revealed that IVM synergistically improved the tumor growth inhibition of mEHT after the fourth treatment. Based on the Bliss independence principle,^41^ the observed interaction between mEHT and IVM was synergism. The observed tumor inhibition rate for mEHT + IVM surpassed the Bliss-predicted inhibition rate calculated for the individual effects of IVM and mEHT. Consistent with our findings, previous studies have demonstrated synergistic effects of IVM with various anticancer therapies including chemotherapeutic agents, such as docetaxel and cyclophosphamide in vitro^44^ and gemcitabine in a pancreatic cancer mouse model.^45^ Moreover, IVM synergized with anti-PD1^46^ and sorafenib^47^ in breast and hepatocellular carcinoma mouse models, respectively.

HSPB1 can interfere with cancer therapy by inhibiting cancer cell apoptosis.^48^ In addition, phosphorylated HSPB1 suppresses actin polymerization, preventing cytoskeletal disruption and caspase-3 activation.^48^ In our study, HSPB1 was upregulated 24 h after mEHT. When combined with mEHT, IVM could not affect the gene or protein expression of HSPB1. However, IVM significantly inhibited the phosphorylation of HSPB1 after mEHT treatment. HSPB1 phosphorylation is mediated by p38 MAPK which activates MAPKAP2 kinase to directly phosphorylate HSPB1.^6^ It was reported that IVM did not affect p38 MAPK phosphorylation in a human prostate cancer cell line. However, IVM could inhibit the phosphorylation of HSPB1 in the presence of MAPKAP2, suggesting blockade of MAPKAP2-mediated phosphorylation.^24^ Similar to our results, IVM inhibited HSPB1 phosphorylation, augmenting tumor growth inhibition of paclitaxel in a prostate cancer mouse model.^24^ Additionally, the inhibition of HSPB1 phosphorylation by IVM sensitized ovarian cancer cells to cisplatin treatment.^49^ In the clinic, the inhibition of HSPB1 using an antisense oligonucleotide improved the overall survival of patients with urothelial carcinoma treated with HSPB1 antisense oligo + docetaxel compared to docetaxel alone^50^

In the current study, mEHT-induced tumor damage was further aggravated when mEHT was combined with IVM. The strong tumor damage in the mEHT + IVM group was associated with the strongest pro-apoptotic and antiproliferative effects. In our study, IVM monotherapy did not exert significant pro-apoptotic or antiproliferative effects after 8 days of daily treatment. IVM was reported to inhibit proliferation in a gastric cancer mouse model.^20^ In addition, IVM alone inhibited proliferation and induced apoptosis in mice bearing esophageal squamous cell carcinoma.^23^ However, in those studies,^20,23^ IVM was administered in higher doses (10–25 mg/kg) for 2–5 weeks.

Chemotherapy is the most common treatment of TNBC patients.^51,52^ However, the clinical outcome is influenced by systemic toxicity, which limits the administered dose.^53^ The current study revealed that mEHT + IVM is a well-tolerated treatment combination for TNBC, as the body weight loss did not exceed 6% in any of the treated mice. To evaluate the possible clinical translational potential of the applied dose, we used the body surface area dose conversion method to calculate the human equivalent dose.^54,55^ In our mouse model, the synergism between mEHT and IVM was achieved at a 7.5 mg/kg dose of IVM. This dose is equivalent to a 0.6 mg/kg dose in humans. Clinical studies revealed that 0.6 mg/kg of IVM was well-tolerated.^15,56 −58^ In addition, IVM was not associated with signs of neurotoxicity—a potential side effect of IVM in healthy adults at doses up to 2 mg/kg.^15^ Furthermore, the daily administration of 1 mg/kg IVM was safe in patients with acute myelogenous leukemia.^59^

## 5 Conclusions

In conclusion, the present study demonstrated for the first time that the inhibition of mEHT-induced HSPs can be achieved by FDA-approved IVM. The molecular mechanism of this synergism was the inhibition of mEHT-induced HSPB1 phosphorylation. Consequently, a combination of IVM and mEHT induced the strongest tumor damage, coupled with the strongest pro-apoptotic and antiproliferative activity. Importantly, the synergy between mEHT and IVM was attained at a well-tolerable and clinically relevant dose, supporting further clinical investigation of this combination in TNBC patients.

## Data Availability

All data associated with this study are presented in the paper. The data that support the findings of this study are available from the corresponding author upon reasonable request.

## Supporting Information Available

PCR analysis for gene expression of progesterone receptor (PR), estrogen receptor (ER), and human epidermal growth factor receptor 2 (HER-2) in normal mouse mammary gland tissue, 4T1 cells, and 4T1 tumors (PDF)

## Notes

The studies did not involve human participants, human data, or human tissue. Interventions and housing of the animals conformed to the Hungarian Laws No. XXVIII/1998 and LXVII/2002 about the protection and welfare of animals and the directives of the EuroEuropean Union. All animal procedures were approved by the National Scientific Ethical Committee on Animal Experimentation under No. PE/EA/50-2/2019.

## Notes

The manuscript does not contain any individual person’s data in any form (including individual details, images or videos).

## Abbreviations

TNBC: triple-negative breast cancer
mEHT: modulated electro-hyperthermia
HSPB1: heat shock protein beta 1
IVM: ivermectin ER estrogen receptor
PR: progesterone receptor
HER2: human epidermal growth factor receptor-2
Ser: serine (Ser) residues
cC3: cleaved caspase 3
Ki67: Kiel proliferation marker
US: ultrasound
cal: caliper
FFPE: formalin-fixed paraffin-embedded
IHC: immunohistochemistry
WB: Western blot
H&E: hematoxylin-eosin staining

## References

[ref1] Dass S. A.; Tan K. L.; Selva Rajan R.; Mokhtar N. F.; Mohd Adzmi E. R.; Wan Abdul Rahman W. F.; Tengku Din T. A. D. A. A.; Balakrishnan V. Triple Negative Breast Cancer: A Review of Present and Future Diagnostic Modalities. Medicina 2021, 57 (1), 62 10.3390/medicina57010062. 33445543 PMC7826673

[ref2] Mehanna J.; Haddad F. G.; Eid R.; Lambertini M.; Kourie H. R. Triple-negative breast cancer: Current perspective on the evolving therapeutic landscape. Int. J. Women’s Health 2019, 11, 431–437. 10.2147/IJWH.S178349. 31447592 PMC6682754

[ref3] Matsumoto T.; Urushido M.; Ide H.; Ishihara M.; Hamada-Ode K.; Shimamura Y.; Ogata K.; Inoue K.; Taniguchi Y.; Taguchi T.; et al. Small Heat Shock Protein Beta-1 (HSPB1) Is Upregulated and Regulates Autophagy and Apoptosis of Renal Tubular Cells in Acute Kidney Injury. PLoS One 2015, 10 (5), e0126229 10.1371/journal.pone.0126229. 25962073 PMC4427334

[ref4] Abisambra J. F.; Jinwal U. K.; Jones J. R.; Blair L. J.; Koren J. 3rd; Dickey C. A. Exploiting the diversity of the heat-shock protein family for primary and secondary tauopathy therapeutics. Curr. Neuropharmacol. 2011, 9 (4), 623–631. 10.2174/157015911798376226. 22654720 PMC3263456

[ref5] McDonald E. T.; Bortolus M.; Koteiche H. A.; McHaourab H. S. Sequence, structure, and dynamic determinants of Hsp27 (HspB1) equilibrium dissociation are encoded by the N-terminal domain. Biochemistry 2012, 51 (6), 1257–1268. 10.1021/bi2017624. 22264079 PMC3293247

[ref6] Kostenko S.; Moens U. Heat shock protein 27 phosphorylation: Kinases, phosphatases, functions and pathology. Cell. Mol. Life Sci. 2009, 66 (20), 3289–3307. 10.1007/s00018-009-0086-3. 19593530 PMC11115724

[ref7] Kim Y. J.; Shuman J.; Sette M.; Przybyla A. Nuclear localization and phosphorylation of three 25-kilodalton rat stress proteins. Mol. Cell. Biol. 1984, 4 (3), 468–474. 10.1128/mcb.4.3.468-474.1984. 6717429 PMC368724

[ref8] Lavoie J. N.; Gingras-Breton G.; Tanguay R. M.; Landry J. Induction of Chinese hamster HSP27 gene expression in mouse cells confers resistance to heat shock. HSP27 stabilization of the microfilament organization. J. Biol. Chem. 1993, 268 (5), 3420–3429. 10.1016/S0021-9258(18)53711-X. 8429018

[ref9] Loktionova S. A.; Ilyinskaya O. P.; Gabai V. L.; Kabakov A. E. Distinct effects of heat shock and ATP depletion on distribution and isoform patterns of human Hsp27 in endothelial cells. FEBS Lett. 1996, 392 (2), 100–104. 10.1016/0014-5793(96)00792-2. 8772183

[ref10] Geum D.; Son G. H.; Kim K. Phosphorylation-dependent Cellular Localization and Thermoprotective Role of Heat Shock Protein 25 in Hippocampal Progenitor Cells*. J. Biol. Chem. 2002, 277 (22), 19913–19921. 10.1074/jbc.M104396200. 11912188

[ref11] Lavoie J. N.; Lambert H.; Hickey E.; Weber L. A.; Landry J. Modulation of Cellular Thermoresistance and Actin Filament Stability Accompanies Phosphorylation-Induced Changes in the Oligomeric Structure of Heat Shock Protein 27. Mol. Cell. Biol. 1995, 15 (1), 505–516. 10.1128/MCB.15.1.505. 7799959 PMC232001

[ref12] Huo Q.; Wang J.; Xie N. High HSPB1 expression predicts poor clinical outcomes and correlates with breast cancer metastasis. BMC Cancer 2023, 23 (1), 501 10.1186/s12885-023-10983-3. 37268925 PMC10239126

[ref13] Crump A. Ivermectin: Enigmatic multifaceted’wonder’ drug continues to surprise and exceed expectations. J. Antibiot. 2017, 70 (5), 495–505. 10.1038/ja.2017.11. 28196978

[ref14] Löscher W. Is the antiparasitic drug ivermectin a suitable candidate for the treatment of epilepsy?. Epilepsia 2023, 64 (3), 553–566. 10.1111/epi.17511. 36645121

[ref15] Guzzo C. A.; Furtek C. I.; Porras A. G.; Chen C.; Tipping R.; Clineschmidt C. M.; Sciberras D. G.; Hsieh J. Y.; Lasseter K. C. Safety, tolerability, and pharmacokinetics of escalating high doses of ivermectin in healthy adult subjects. J. Clin. Pharmacol. 2002, 42 (10), 1122–1133. 10.1177/009127002401382731. 12362927

[ref16] Porubcin S.; Rovnakova A.; Zahornacky O.; Jarcuska P. Intravenous veterinary ivermectin in a COVID-19 patient causing neurotoxicity. IDCases 2022, 27, e01446 10.1016/j.idcr.2022.e01446. 35155125 PMC8818555

[ref17] Yang C. C. Acute human toxicity of macrocyclic lactones. Curr. Pharm. Biotechnol. 2012, 13 (6), 999–1003. 10.2174/138920112800399059. 22039794

[ref18] Kwon Y. J.; Petrie K.; Leibovitch B. A.; Zeng L.; Mezei M.; Howell L.; Gil V.; Christova R.; Bansal N.; Yang S.; Sharma R.; Ariztia E. V.; Frankum J.; Brough R.; Sbirkov Y.; Ashworth A.; Lord C. J.; Zelent A.; Farias E.; Zhou M. M.; Waxman S. Selective Inhibition of SIN3 Corepressor with Avermectins as a Novel Therapeutic Strategy in Triple-Negative Breast Cancer. Mol. Cancer Ther. 2015, 14 (8), 1824–1836. 10.1158/1535-7163.MCT-14-0980-T. 26078298 PMC4529816

[ref19] Draganov D.; Gopalakrishna-Pillai S.; Chen Y. R.; Zuckerman N.; Moeller S.; Wang C.; Ann D.; Lee P. P. Modulation of P2 × 4/P2 × 7/Pannexin-1 sensitivity to extracellular ATP via Ivermectin induces a non-apoptotic and inflammatory form of cancer cell death. Sci. Rep. 2015, 5, 16222 10.1038/srep16222. 26552848 PMC4639773

[ref20] Nambara S.; Masuda T.; Nishio M.; Kuramitsu S.; Tobo T.; Ogawa Y.; Hu Q.; Iguchi T.; Kuroda Y.; Ito S.; Eguchi H.; Sugimachi K.; Saeki H.; Oki E.; Maehara Y.; Suzuki A.; Mimori K. Antitumor effects of the antiparasitic agent ivermectin via inhibition of Yes-associated protein 1 expression in gastric cancer. Oncotarget 2017, 8 (64), 107666–107677. 10.18632/oncotarget.22587. 29296196 PMC5746098

[ref21] Zhang P.; Zhang Y.; Liu K.; Liu B.; Xu W.; Gao J.; Ding L.; Tao L. Ivermectin induces cell cycle arrest and apoptosis of HeLa cells via mitochondrial pathway. Cell Proliferation 2019, 52 (2), e12543 10.1111/cpr.12543. 30515909 PMC6496724

[ref22] Song D.; Liang H.; Qu B.; Li Y.; Liu J.; Zhang Y.; Li L.; Hu L.; Zhang X.; Gao A. Ivermectin inhibits the growth of glioma cells by inducing cell cycle arrest and apoptosis in vitro and in vivo. J. Cell. Biochem. 2019, 120 (1), 622–633. 10.1002/jcb.27420. 30596403

[ref23] Xu N.; Lu M.; Wang J.; Li Y.; Yang X.; Wei X.; Si J.; Han J.; Yao X.; Zhang J.; Liu J.; Li Y.; Yang H.; Bao D. Ivermectin induces apoptosis of esophageal squamous cell carcinoma via mitochondrial pathway. BMC Cancer 2021, 21 (1), 1307 10.1186/s12885-021-09021-x. 34876051 PMC8650430

[ref24] Nappi L.; Aguda A. H.; Nakouzi N. A.; Lelj-Garolla B.; Beraldi E.; Lallous N.; Thi M.; Moore S.; Fazli L.; Battsogt D.; Stief S.; Ban F.; Nguyen N. T.; Saxena N.; Dueva E.; Zhang F.; Yamazaki T.; Zoubeidi A.; Cherkasov A.; Brayer G. D.; Gleave M. Ivermectin inhibits HSP27 and potentiates efficacy of oncogene targeting in tumor models. J. Clin. Invest. 2019, 130 (2), 699–714. 10.1172/JCI130819. PMC6994194 31845908

[ref25] Nagata T.; Kanamori M.; Sekine S.; Arai M.; Moriyama M.; Fujii T. Clinical study of modulated electro-hyperthermia for advanced metastatic breast cancer. Mol. Clin. Oncol. 2021, 14 (5), 103 10.3892/mco.2021.2265. 33796292 PMC8010507

[ref26] Kim S.; Lee J. H.; Cha J.; You S. H. Beneficial effects of modulated electro-hyperthermia during neoadjuvant treatment for locally advanced rectal cancer. Int. J. Hyperthermia 2021, 38 (1), 144–151. 10.1080/02656736.2021.1877837. 33557636

[ref27] Fiorentini G.; Sarti D.; Casadei V.; Milandri C.; Dentico P.; Mambrini A.; Nani R.; Fiorentini C.; Guadagni S. Modulated Electro-Hyperthermia as Palliative Treatment for Pancreatic Cancer: A Retrospective Observational Study on 106 Patients. Integr. Cancer Ther. 2019, 18, 1534735419878505 10.1177/1534735419878505. 31561722 PMC6767725

[ref28] Minnaar C. A.; Maposa I.; Kotzen J. A.; Baeyens A. Effects of Modulated Electro-Hyperthermia (mEHT) on Two and Three Year Survival of Locally Advanced Cervical Cancer Patients. Cancers 2022, 14 (3), 656 10.3390/cancers14030656. 35158924 PMC8833695

[ref29] Fiorentini G.; Szasz A. Hyperthermia today: Electric energy, a new opportunity in cancer treatment. J. Cancer Res. Ther. 2006, 2 (2), 41–46. 10.4103/0973-1482.25848. 17998673

[ref30] Aloss K.; Hamar P. Augmentation of the EPR effect by mild hyperthermia to improve nanoparticle delivery to the tumor. Biochim. Biophys. Acta, Rev. Cancer 2024, 1879 (4), 189109 10.1016/j.bbcan.2024.189109. 38750699

[ref31] Vander Heiden M. G.; Cantley L. C.; Thompson C. B. Understanding the Warburg effect: The metabolic requirements of cell proliferation. Science 2009, 324 (5930), 1029–1033. 10.1126/science.1160809. 19460998 PMC2849637

[ref32] Giunashvili N.; Thomas J. M.; Schvarcz C. A.; Viana P. H. L.; Aloss K.; Bokhari S. M. Z.; Koós Z.; Bócsi D.; Major E.; Balogh A.; Benyó Z.; Hamar P. Enhancing therapeutic efficacy in triple-negative breast cancer and melanoma: Synergistic effects of modulated electro-hyperthermia (mEHT) with NSAIDs especially COX-2 inhibition in in vivo models. Mol. Oncol. 2024, 18, 1012 10.1002/1878-0261.13585. 38217262 PMC10994232

[ref33] Lee S.-Y.; Lorant G.; Grand L.; Szasz A. M. The Clinical Validation of Modulated Electro-Hyperthermia (mEHT). Cancers 2023, 15, 4569 10.3390/cancers15184569. 37760538 PMC10526385

[ref34] Bokhari S. M. Z.; Aloss K.; Leroy Viana P. H.; Schvarcz C. A.; Besztercei B.; Giunashvili N.; Bócsi D.; Koós Z.; Balogh A.; et al. Digoxin-Mediated Inhibition of Potential Hypoxia-Related Angiogenic Repair in Modulated Electro-Hyperthermia (mEHT)-Treated Murine Triple-Negative Breast Cancer Model. ACS Pharmacol. Transl. Sci. 2024, 7, 456–466. 10.1021/acsptsci.3c00296. 38357275 PMC10863435

[ref35] Viana P.; Hamar P. Targeting the heat shock response induced by modulated electro-hyperthermia (mEHT) in cancer. Biochim. Biophys. Acta, Rev. Cancer 2024, 1879 (2), 189069 10.1016/j.bbcan.2023.189069. 38176599

[ref36] Danics L.; Schvarcz C. A.; Viana P.; Vancsik T.; Krenács T.; Benyó Z.; Kaucsár T.; Hamar P. Exhaustion of Protective Heat Shock Response Induces Significant Tumor Damage by Apoptosis after Modulated Electro-Hyperthermia Treatment of Triple Negative Breast Cancer Isografts in Mice. Cancers 2020, 12 (9), 2581 10.3390/cancers12092581. 32927720 PMC7565562

[ref37] Viana P. H. L.; Schvarcz C. A.; Danics L. O.; Besztercei B.; Aloss K.; Bokhari S. M. Z.; Giunashvili N.; Bócsi D.; Koós Z.; Benyó Z.; Hamar P. Heat shock factor 1 inhibition enhances the effects of modulated electro hyperthermia in a triple negative breast cancer mouse model. Sci. Rep. 2024, 14 (1), 8241 10.1038/s41598-024-57659-x. 38589452 PMC11002009

[ref38] Wang R. E.; Kao J. L.; Hilliard C. A.; Pandita R. K.; Roti Roti J. L.; Hunt C. R.; Taylor J. S. Inhibition of heat shock induction of heat shock protein 70 and enhancement of heat shock protein 27 phosphorylation by quercetin derivatives. J. Med. Chem. 2009, 52 (7), 1912–1921. 10.1021/jm801445c. 19296652 PMC2763579

[ref39] Schvarcz C. A.; Danics L.; Krenács T.; Viana P.; Béres R.; Vancsik T.; Nagy Á.; Gyenesei A.; Kun J.; Fonović M.; Vidmar R.; Benyó Z.; Kaucsár T.; Hamar P. Modulated Electro-Hyperthermia Induces a Prominent Local Stress Response and Growth Inhibition in Mouse Breast Cancer Isografts. Cancers 2021, 13 (7), 1744 10.3390/cancers13071744. 33917524 PMC8038813

[ref40] Aloss K.; Bokhari S. M.; Leroy Viana P. H.; Giunashvili N.; Schvarcz C. A.; Szénási G.; Bócsi D.; Koós Z.; Storm G.; Miklós Z.; Benyó Z.; Hamar P. Modulated Electro-Hyperthermia Accelerates Tumor Delivery and Improves Anticancer Activity of Doxorubicin Encapsulated in Lyso-Thermosensitive Liposomes in 4T1-Tumor-Bearing Mice. Int. J. Mol. Sci. 2024, 25, 3101 10.3390/ijms25063101. 38542073 PMC10970314

[ref41] Liu Q.; Yin X.; Languino L. R.; Altieri D. C. Evaluation of drug combination effect using a Bliss independence dose-response surface model. Stat. Biopharm. Res. 2018, 10 (2), 112–122. 10.1080/19466315.2018.1437071. 30881603 PMC6415926

[ref42] Rio D. C.; Ares M.; Hannon G. J.; Nilsen T. W. Purification of RNA using TRIzol (TRI reagent) CSHL Press, Cold Spring Harbor NY, USA 2010 10.1101/pdb.prot5439 20516177

[ref43] Vancsik T.; Kovago C.; Kiss E.; Papp E.; Forika G.; Benyo Z.; Meggyeshazi N.; Krenacs T. Modulated electro-hyperthermia induced loco-regional and systemic tumor destruction in colorectal cancer allografts. J. Cancer 2018, 9 (1), 41–53. 10.7150/jca.21520. 29290768 PMC5743710

[ref44] Juarez M.; Schcolnik-Cabrera A.; Dominguez-Gomez G.; Chavez-Blanco A.; Diaz-Chavez J.; Duenas-Gonzalez A. Antitumor effects of ivermectin at clinically feasible concentrations support its clinical development as a repositioned cancer drug. Cancer Chemother. Pharmacol. 2020, 85 (6), 1153–1163. 10.1007/s00280-020-04041-z. 32474842

[ref45] Lee D. E.; Kang H. W.; Kim S. Y.; Kim M. J.; Jeong J. W.; Hong W. C.; Fang S.; Kim H. S.; Lee Y. S.; Kim H. J.; Park J. S. Ivermectin and gemcitabine combination treatment induces apoptosis of pancreatic cancer cells via mitochondrial dysfunction. Front. Pharmacol. 2022, 13, 934746 10.3389/fphar.2022.934746. 36091811 PMC9459089

[ref46] Draganov D.; Han Z.; Rana A.; Bennett N.; Irvine D. J.; Lee P. P. Ivermectin converts cold tumors hot and synergizes with immune checkpoint blockade for treatment of breast cancer. Npj Breast Cancer 2021, 7 (1), 22 10.1038/s41523-021-00229-5. 33654071 PMC7925581

[ref47] Lu H.; Zhou L.; Zuo H.; Le W.; Hu J.; Zhang T.; Li M.; Yuan Y. Ivermectin synergizes sorafenib in hepatocellular carcinoma via targeting multiple oncogenic pathways. Pharmacol. Res. Perspect. 2022, 10 (3), e00954 10.1002/prp2.954. 35568994 PMC9107598

[ref48] Kennedy D.; Jäger R.; Mosser D. D.; Samali A. Regulation of apoptosis by heat shock proteins. IUBMB Life 2014, 66 (5), 327–338. 10.1002/iub.1274. 24861574

[ref49] Heiserman J. P.; Nallanthighal S.; Gifford C. C.; Graham K.; Samarakoon R.; Gao C.; Sage J. J.; Zhang W.; Higgins P. J.; Cheon D. J. Heat Shock Protein 27, a Novel Downstream Target of Collagen Type XI alpha 1, Synergizes with Fatty Acid Oxidation to Confer Cisplatin Resistance in Ovarian Cancer Cells. Cancers 2021, 13 (19), 4855 10.3390/cancers13194855. 34638339 PMC8508313

[ref50] Rosenberg J. E.; Hahn N. M.; Regan M. M.; Werner L.; Alva A.; George S.; Picus J.; Alter R.; Balar A.; Hoffman-Censits J.; Grivas P.; Lauer R.; Guancial E. A.; Hoimes C.; Sonpavde G.; Albany C.; Stein M. N.; Breen T.; Jacobs C.; Anderson K.; Bellmunt J.; Lalani A.-K. A.; Pal S.; Choueiri T. K. Apatorsen plus docetaxel versus docetaxel alone in platinum-resistant metastatic urothelial carcinoma (Borealis-2). Br. J. Cancer 2018, 118 (11), 1434–1441. 10.1038/s41416-018-0087-9. 29765151 PMC5988804

[ref51] Furlanetto J.; Loibl S. Optimal Systemic Treatment for Early Triple-Negative Breast Cancer. Breast Care 2020, 15 (3), 217–226. 10.1159/000508759. 32774215 PMC7383279

[ref52] Wahba H. A.; El-Hadaad H. A. Current approaches in treatment of triple-negative breast cancer. Cancer Biol. Med. 2015, 12 (2), 106–116. 10.7497/j.issn.2095-3941.2015.0030. 26175926 PMC4493381

[ref53] Aloss K.; Hamar P. Recent Preclinical and Clinical Progress in Liposomal Doxorubicin. Pharmaceutics 2023, 15, 893 10.3390/pharmaceutics15030893. 36986754 PMC10054554

[ref54] Nair A. B.; Jacob S. A simple practice guide for dose conversion between animals and human. J. Basic Clin. Pharm. 2016, 7 (2), 27–31. 10.4103/0976-0105.177703. 27057123 PMC4804402

[ref55] Food and Drug Administration, Estimating the Maximum Safe Starting Dose in Initial Clinical Trials for Therapeutics in Adult Healthy Volunteers Food and Drug Administration 2005.

[ref56] Smit M. R.; Ochomo E. O.; Aljayyoussi G.; Kwambai T. K.; Abong’o B. O.; Chen T.; Bousema T.; Slater H. C.; Waterhouse D.; Bayoh N. M.; Gimnig J. E.; Samuels A. M.; Desai M. R.; Phillips-Howard P. A.; Kariuki S. K.; Wang D.; Ward S. A.; ter Kuile F. O. Safety and mosquitocidal efficacy of high-dose ivermectin when co-administered with dihydroartemisinin-piperaquine in Kenyan adults with uncomplicated malaria (IVERMAL): A randomised, double-blind, placebo-controlled trial. Lancet Infect. Dis. 2018, 18 (6), 615–626. 10.1016/S1473-3099(18)30163-4. 29602751

[ref57] Awadzi K.; Opoku N. O.; Addy E. T.; Quartey B. T. The chemotherapy of onchocerciasis. XIX: The clinical and laboratory tolerance of high dose ivermectin. Trop Med. Parasitol. 1995, 46 (2), 131–137. 8525285

[ref58] Navarro M.; Camprubí D.; Requena-Méndez A.; Buonfrate D.; Giorli G.; Kamgno J.; Gardon J.; Boussinesq M.; Muñoz J.; Krolewiecki A. Safety of high-dose ivermectin: A systematic review and meta-analysis. J. Antimicrob. Chemother. 2020, 75 (4), 827–834. 10.1093/jac/dkz524. 31960060

[ref59] Gregianin L. J.; Burger J. A. Continuous high-dose ivermectin appears to be safe in patients with acute myelogenous leukemia and could inform clinical repurposing for COVID-19 infection. Leuk. Lymphoma 2020, 61 (10), 2536–2537. 10.1080/10428194.2020.1786559. 32611256

