# Supplementary material for: Ivermectin Synergizes with Modulated Electro-hyperthermia and Improves Its Anticancer Effects in a Triple-Negative Breast Cancer Mouse Model
Source: ACS Pharmacol Transl Sci. 2024 Jul 17;7(8):2496–506. doi: 10.1021/acsptsci.4c00314 (PMC11320741; doi:10.1021/acsptsci.4c00314)
Supplement: Supplementary file 1 — pt4c00314_si_001.pdf [file pt4c00314_si_001.pdf]

Supporting Information

Ivermectin synergizes with modulated electro-hyperthermia and improves its anti-cancer effects in a triple-negative breast cancer mouse model

Kenan Aloss[1, 2], Pedro Henrique Leroy Viana[1], Syeda Mahak Zahra Bokhari[1], Nino Giunashvili[1], Csaba András Schvarcz[1, 3], Dániel Bócsi[1], Zoltán Koós[1], Zoltán Benyó[1, 3], Péter Hamar[1*]

[1] Institute of Translational Medicine, Semmelweis University, Üllői út 26., Budapest, 1085, Hungary.
[2] Department of Pharmacology and Pharmacotherapy, Semmelweis University, Budapest, Hungary.
[3] HUN-REN-SU Cerebrovascular and Neurocognitive Diseases Research Group, Budapest, Hungary, Tűzoltó utca 37-47., Budapest, 1094, Hungary.

*Email: hamar.peter@semmelweis.hu

*Phone number: +36208259751

*Fax number: (+36 20) 825 6507

*Postal address: 1428 Budapest, Pf. 2.

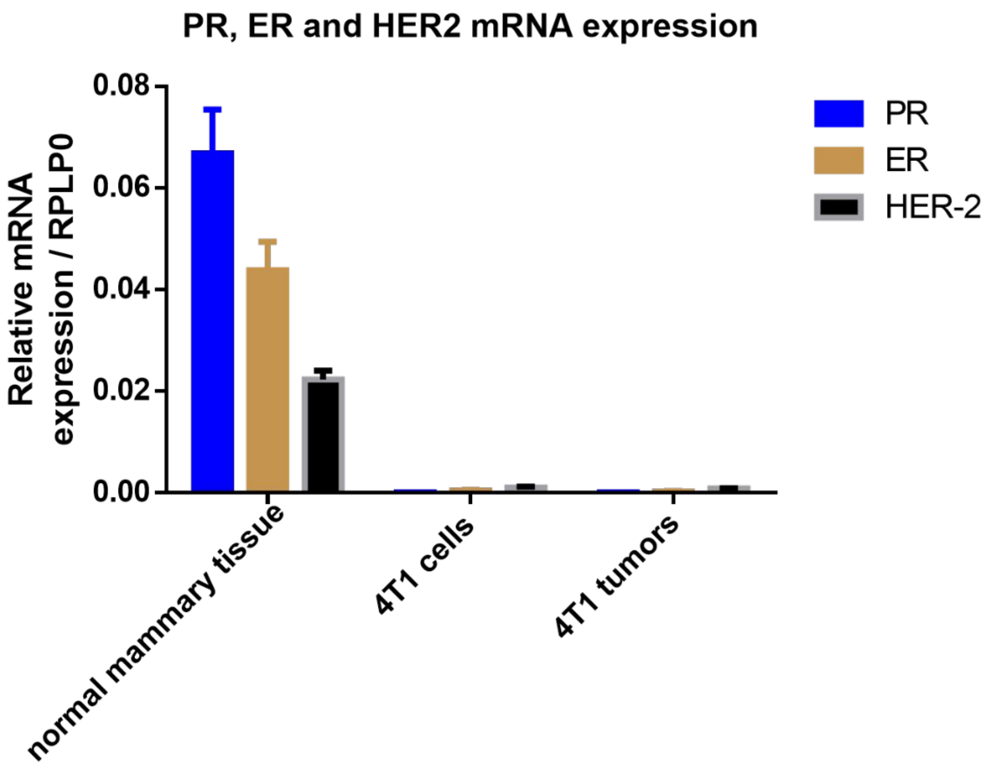

Figure S1. Gene expression of progesterone receptor (PR), estrogen receptor (ER), and human epidermal growth factor receptor 2 (HER-2) in normal mouse mammary gland tissue, 4T1 cells, and 4T1 tumors
